# The Role of ADCY1 in Regulating the Sensitivity of Platinum-Based Chemotherapy in NSCLC

**DOI:** 10.3390/ph17091118

**Published:** 2024-08-24

**Authors:** Ting Zou, Jun-Yan Liu, Zhao-Qian Liu, Di Xiao, Juan Chen

**Affiliations:** 1 Department of Pharmacy, National Institution of Drug Clinical Trial, Xiangya Hospital, Central South University, Changsha 410008, China; zouting@csu.edu.cn; 2 Hunan Key Laboratory of Pharmacogenetics, Department of Clinical Pharmacology, Xiangya Hospital, Central South University, Changsha 410008, China; zqliu@csu.edu.cn; 3 National Clinical Research Center for Geriatric Disorders, Xiangya Hospital, Central South University, Changsha 410008, China; 4 Department of Orthopaedics, Xiangya Hospital, Central South University, Changsha 410008, China; liujunyana@126.com; 5 The Hunan Institute of Pharmacy Practice and Clinical Research, Xiangya Hospital, Central South University, Changsha 410008, China

**Keywords:** ADCY1, drug resistance, lung cancer, platinum-based chemotherapy

## Abstract

Lung cancer has the highest fatality rate among malignant tumors in the world. Finding new biomarkers of drug resistance is of great importance in the prognosis of lung cancer patients. We found that the polymorphisms of Adenylate Cyclase 1 (ADCY1) are significantly associated with platinum-based chemotherapy resistance in lung cancer patients in our previous research. In this study, we wanted to identify the mechanism of ADCY1 affecting platinum resistance. We used an MTT assay to find if the expression of ADCY1 is associated with the sensitivity of cisplatin in A549, H1299, and A549-DDP cells. Then, we performed CCK-8 tests to detect the absorbance of these cells stimulated by ADCY1, which can discover the cell proliferation that is affected by ADCY1. We investigated cell apoptosis and cell cycles regulated by ADCY1 through the flow cytometry assay. RNA sequencing was used to find the downstream genes affected by ADCY1 which may be associated with drug resistance in lung cancer patients. ADCY1 has higher expression in lung cancer cells than in normal cells. ADCY1 can affect cisplatin resistance in lung cancer cells by regulating cell proliferation, cell apoptosis, and the cell cycle. It may control cell apoptosis by regulating the classical apoptosis biomarkers Bax and Bcl2. Our study showed that ADCY1 may be a new biomarker in the prognosis of lung cancer patients. Much work remains to be carried out to clarify the mechanism in this important emerging field.

## 1. Introduction

Recent studies indicate that lung cancer remains the main cause of cancer mortality around the world, accounting for 18.4% of all cancer patients [1]. Lung cancer is one of the most important diseases and the related therapies have been widely studied [2,3]. It has been divided into two types, including non-small cell lung cancer (NSCLC) and small cell lung cancer (SCLC), and NSCLC consists of lung adenocarcinoma, lung squamous cell carcinoma, and large cell lung cancer [4]. It is well known that the risk factors include smoking status, living environment, occupation, and so on [5]. Although the incidence rate of lung cancer has decreased slightly in recent years, the 5-year survival rate of lung cancer is about 19% and the prognosis is still unsatisfactory [6,7].

Although there are many other treatments emerging in an endless stream, platinum-based chemotherapy is still the first-line therapy for lung cancer patients [8]. Chemotherapy can also recover immune surveillance, which can enhance the curative effects [9]. The action of cisplatin is to form DNA adducts in DNA strands or between DNA strands by interacting with purines on DNA [10]. Specific cells recognize the adducts as DNA damage and then activate DNA repair mechanisms, such as nucleotide excision repair, mismatch repair, base excision repair, non-homologous end joining, and homologous recombination [11]. Platinum-based chemotherapy usually has an effective initial response, but acquired resistance affects the sensitivity of chemotherapy in lung cancer patients [12]. According to relevant evidence, the possible inducements of platinum-based chemotherapy resistance include DNA damage increases, drug accumulation decreases, detoxification, epigenetic changes, apoptosis decreases, and membrane transport variation, as well as other possible mechanisms [13].

Cisplatin resistance is related to many factors, especially in DNA damage repair or the reaction with metallothionein and glutathione to inactive drugs [14]. Combination therapeutics can reduce the side effects and drug resistance of cisplatin, which also can minimize the interference of antitumor activity [15]. Epithelial–mesenchymal transition (EMT) and multidrug resistance can influence the sensitivity of platinum-based chemotherapy [16]. The increase in DNA repair is a prominent feature of platinum-resistant cells [17]. Platinum can adduct with DNA strands, which may inhibit DNA replication and transcription; it can also lead to DNA double-strand breaks, which may be the mechanism of platinum killing tumor cells [18]. DNA repair failure will lead to cell apoptosis, which means targeting DNA repair is an important tumor therapy [19]. In addition, cisplatin can also block the cell cycle and inhibit cell proliferation [20].

ADCY1 belongs to adenylate cyclase, which is responsible for the synthesis of cAMP’s second messenger, catalyzing adenosine triphosphate (ATP) to cyclic 3′, 5′-monophosphate (cAMP), and releasing pyrophosphate [21,22]. ADCY1 is a transmembrane protein that is stimulated by Ca^2+^/calmodulin and activated by the suppressor of zeste 12 homolog (SUZ12) [23]. ADCY1 acts as an intercourse, converts extracellular signal stimuli into intracellular signals, integrates different signals [24], and causes variation in the cellular microenvironment by regulating cAMP second messengers, resulting in a series of physiological effects, including cellular responses such as cell growth, cell differentiation, cell proliferation, cell apoptosis, and cell metabolism [25,26]. cAMP signaling can also regulate tumor cell apoptosis induced by γ-ray and anticancer drugs [27].

According to recent studies, ADCY1 in tumor drug resistance has rarely been reported, mainly related to tumor occurrence, development, and prognosis [28]. ADCY1 is highly expressed in multidrug-resistant Eca-1 cells, and activation of PKA signaling may confer resistance to most chemotherapeutic drugs in esophageal carcinoma cells [29]. ADCY1 was down-regulated in rectal adenocarcinoma metastasis and may play a crucial part in pancreatic secretion and cell adhesion molecular pathways [30]. ADCY1 methylation status may affect the prognosis of glioblastoma patients [31,32]. The expression level of ADCY1 significantly affects the overall survival of patients with renal clear cell tumors or patients with metastatic melanoma [33,34]. ADCY1 is down-regulated in prostate cancer and osteosarcoma and may be involved in the development of prostate cancer and osteosarcoma [35,36].

Our research was based on the whole exome sequencing of the blood samples of 17 patients with non-small cell lung cancer with extreme drug sensitivity receiving platinum-based chemotherapy and 17 patients with non-small cell lung cancer with extreme drug resistance receiving platinum-based chemotherapy. The results were clinically validated. The results revealed that ADCY1 polymorphism rs2293106 (c.3090G>A) was significantly more effective in non-small cell lung cancer patients with platinum-based combined chemotherapy, and the patients with the A allele were more affected than the patients with the G allele who received platinum-based combined chemotherapy [37]. The goal of our study was to investigate the effect of ADCY1 on platinum resistance and find new biomarkers for lung cancer treatment, which may contribute to more intensive guidance in clinical diagnosis and treatment in lung cancer patients.

## 2. Results

### 2.1. The Expression of ADCY1 in Lung Cancer Patients from the Data in TCGA Database

**Figure 1:**
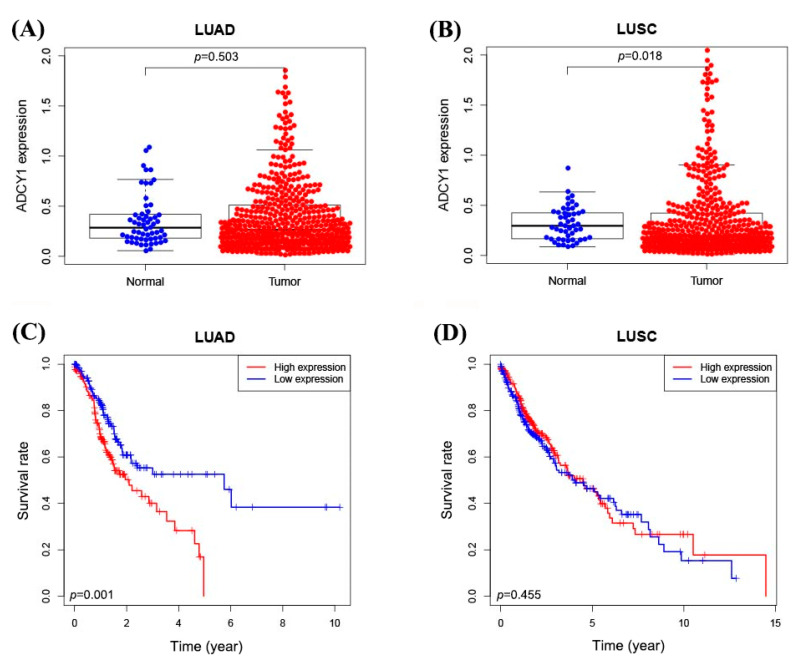
The expression of ADCY1 in lung cancer patients from the data in the TCGA database. (**A**) The expression of ADCY1 in 497 lung adenocarcinoma tissues and 54 normal tissues. (**B**) The expression of ADCY1 in 502 cases of lung squamous cell carcinoma tissues and 49 cases of normal tissues. (**C**) Kaplan–Meier survival curve of ADCY1 expression in lung adenocarcinoma patients. (**D**) Kaplan–Meier survival curve of ADCY1 expression in patients with lung squamous cell carcinoma.

To determine whether the expression of ADCY1 differs between tumor and normal tissues, we searched the RNA sequencing data in the cancer genome atlas (TCGA) database and analyzed the expression differences in the ADCY1 gene in lung cancer patients. As shown in Figure 1, we used the gene expression data of 497 lung adenocarcinoma tissue samples and 502 lung squamous cell carcinoma tissue samples, and found that the expression level of ADCY1 in lung squamous cell carcinoma tissues was higher than that in normal tissues (*p* = 0.018). However, there was no significant difference in the paired comparison in lung adenocarcinoma patients (*p* = 0.503). The higher expression of ADCY1 has a poorer prognosis than the lower expression of ADCY1 in adenocarcinoma of lung cancer patients (*p* = 0.001). Through the data in the TCGA, we found that ADCY1 was overexpressed in lung squamous cell carcinoma, and it is significantly associated with the survival of lung adenocarcinoma. This indicated that ADCY1 may be a potential biomarker in lung cancer treatment.

### 2.2. The Regulation of ADCY1 in the Sensitivity to Cisplatin in Lung Cancer Cells

#### 2.2.1. The Expression of ADCY1 in Different Cells

**Figure 2:**
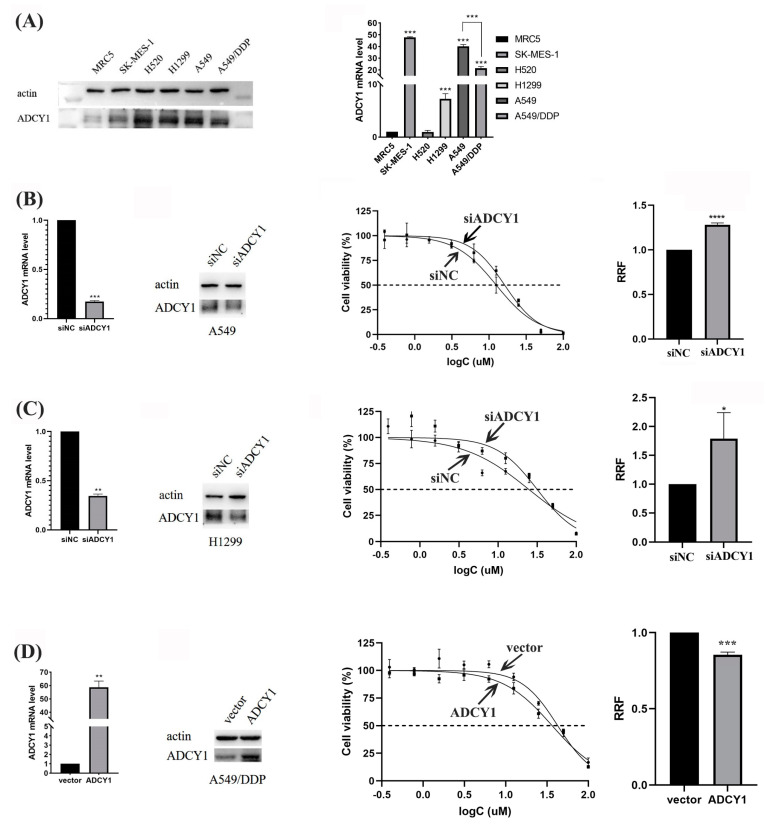
The regulation of ADCY1 in the sensitivity to cisplatin in lung cancer cells. RRF: relative resistance factor. (**A**) The expression of ADCY1 in different cell lines. The effect of down-regulating the expression of ADCY1 on the sensitivity to cisplatin in (**B**) A549 cells and (**C**) H1299 cells. (**D**) The effect of up-regulating the expression of ADCY1 on the sensitivity to cisplatin in A549−DDP cells. *, *p* < 0.05. **, *p* < 0.01. ***, *p* < 0.001. ****, *p* < 0.0001.

We extracted the protein and RNA in human embryonic lung fibroblast cells (MRC5), lung squamous cell carcinoma cells (SK-MES-1 and H520), lung adenocarcinoma cells (H1299 and A549), and cisplatin-resistant lung adenocarcinoma cells (A549-DDP) to analyze the RNA level of ADCY1 and the protein expression in these cells. We found that the RNA level of ADCY1 and protein expression is significantly higher in lung cancer cells than in MRC5 cells, and it has a lower RNA level of ADCY1 in A549/DDP than in A549 cells (*p* < 0.001). This means that the expression of ADCY1 is diverse between normal cells and lung cancer cells, and it may be associated with cisplatin resistance in lung cancer cells (Figure 2A).

#### 2.2.2. Effect of the Knockdown Expression of ADCY1 on the Sensitivity to Cisplatin in Lung Cancer Cells

To investigate the effect of ADCY1 on the sensitivity of lung cancer to cisplatin, we used siRNA to interfere with the expression of ADCY1 in human lung adenocarcinoma cell lines A549 and H1299. As presented in Figure 2B, C, the knockdown efficiency of ADCY1 in A549 cells and H1299 cells was about 80% and 70%, respectively. The MTT assay demonstrated that transferring by siADCY1 could increase cell survival rate in A549 and H1299 cells treated with cisplatin. The sensitivity to cisplatin was significantly decreased and the IC50 value was significantly increased (*p* < 0.0001) in A549 cells transferred with siADCY1 compared with the siNC control group. Similarly, the siADCY1-transfected H1299 cells showed significantly lower sensitivity to cisplatin, and the IC50 value was significantly increased (*p* < 0.05). This indicates that the low expression level of ADCY1 may increase cisplatin resistance in lung cancer cells.

#### 2.2.3. The Effect of Up-Regulating the Expression of ADCY1 on the Sensitivity to Cisplatin in Lung Cancer Cells

Since we found that knockdown of the expression of ADCY1 can reduce the sensitivity to cisplatin in A549 cells and H1299 cells, we wanted to research the influence of up-regulated ADCY1 in A549/DDP cells on platinum sensitivity. We constructed a plasmid carrying the ADCY1 gene to overexpress the expression of ADCY1 in A549/DDP; the overexpression efficiency of ADCY1 in A549/DDP cells was about 60-fold. As shown in Figure 2D, after transferring the ADCY1 plasmid, the sensitivity of the A549/DDP cell line to cisplatin was enhanced, and the IC50 value was significantly decreased (*p* < 0.001) compared with that of the empty plasmid. We discovered that the high expression of ADCY1 can significantly increase cisplatin sensitivity in cisplatin-resistant lung cancer, which is in accordance with the results revealed by the down-regulation of ADCY1 in lung cancer cells.

### 2.3. Effect of ADCY1 on Cell Proliferation and Apoptosis

#### 2.3.1. Effect of ADCY1 on Cell Proliferation

**Figure 3:**
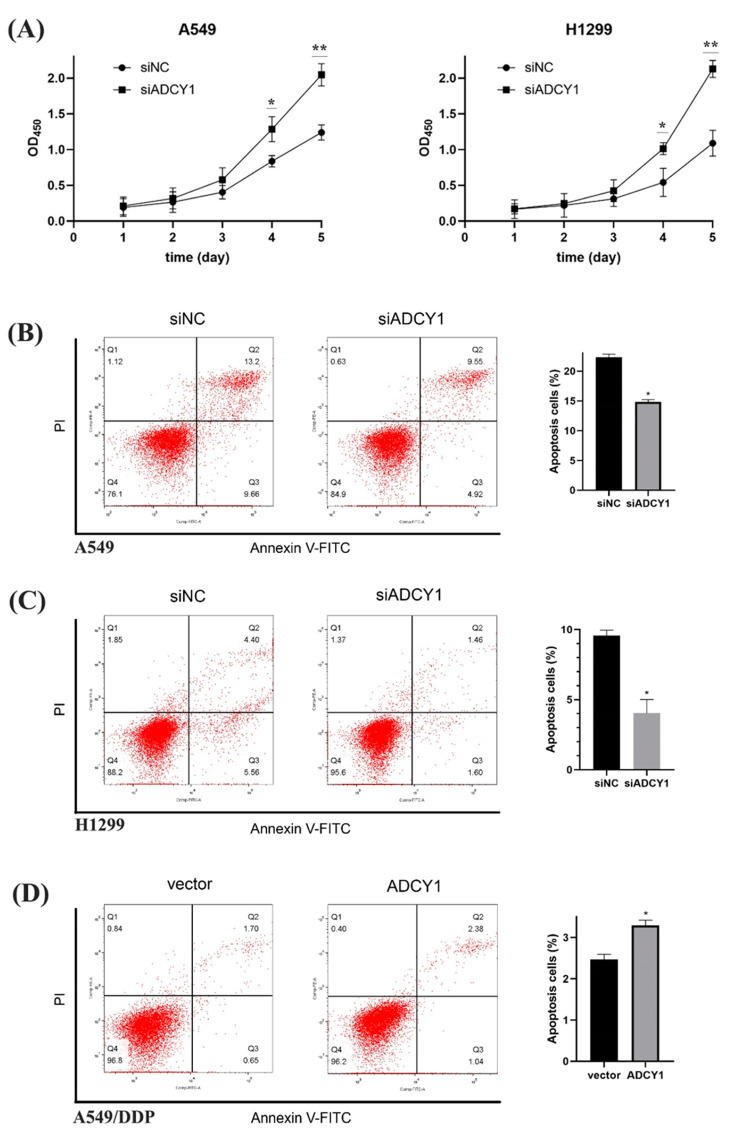
Effect of ADCY1 on cell proliferation and cell apoptosis. (**A**) Effect of ADCY1 on cell proliferation in A549 and H1299 cells. Effect of down-regulating the expression of ADCY1 on cell apoptosis in (**B**) A549 cells and (**C**) H1299 cells. (**D**) Effect of up-regulating the expression of ADCY1 on cell apoptosis in A549-DDP cells. *, *p* < 0.05. **, *p* < 0.01.

As we know, cell proliferation and apoptosis are essential factors in platinum resistance; we wanted to investigate whether ADCY1 plays a role in cell proliferation. After the knockdown of ADCY1 in A549 and H1299 cells, the cells were seeded into 96-well plates, and the absorbance was detected by CCK-8 every day to obtain the growth curve of the cells. The results showed that the OD450 is significantly higher in cells transferred by siADCY1 than the siNC in A549 and H1299 cells, which means that the knockdown of the expression of ADCY1 can accelerate the proliferation of A549 and H1299 cells, and the influence by ADCY1 on cell proliferation began to show from the fourth day (Figure 3A).

#### 2.3.2. Effect of ADCY1 on Cell Apoptosis

We used Annexin V/PI double staining to quantify A549 and H1299 cell apoptosis by flow cytometry. The percentage of specific cell populations at various stages of apoptosis is shown in Figure 3B,C. The apoptosis rate of the A549 control group was 22.35 ± 0.52%, that of the A549 knockdown group was 14.84 ± 0.37%, and there was a significant difference between them. The apoptosis rate of the H1299 control group was 9.58 ± 0.39% and that of the H1299 knockdown group was 4.04 ± 0.98%, which also showed a significant difference, indicating that the knockdown of ADCY1 could inhibit apoptosis caused by cisplatin. As shown in Figure 3D, the apoptosis rate of the control group was 2.47 ± 0.12%, that of the overexpression group was 3.30 ± 0.13%, and there was a significant difference between the two groups using statistical tests, indicating that the overexpression of ADCY1 could promote apoptosis caused by cisplatin.

### 2.4. Effect of ADCY1 on Cell Cycle

**Figure 4:**
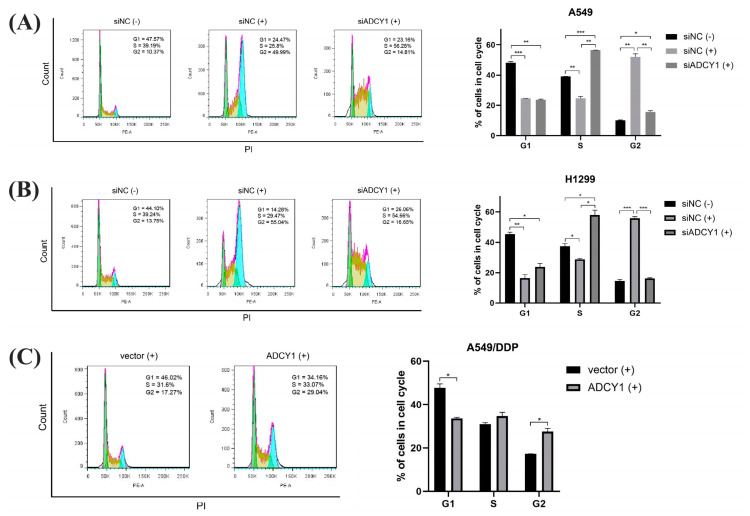
Effect of ADCY1 on cell cycle. Effect of down−regulation of ADCY1 on cell cycle in (**A**) A549 cells and (**B**) H1299 cells. (**C**) Effect of up−regulation of ADCY1 on cell cycle in A549−DDP cells. *, *p* < 0.05. **, *p* < 0.01. ***, *p* < 0.001.

It has been reported that cisplatin can affect the cell cycle and lead to cell cycle arrest, which may inhibit cell proliferation and promote cell apoptosis. After ADCY1 was knocked down in A549 cells and H1299 cells, cisplatin was added and cells were cultured for 48 h. Then, cells were collected and cell cycle distribution was detected by flow cytometry. As shown in Figure 4A,B, the proportion of G2 phase cells in A549 and H1299 cells was significantly increased, resulting in G2 phase arrest and cell proliferation inhibition after cisplatin treatment. Furthermore, compared with the negative control group, the proportion of S-phase cells in A549 and H1299 cells was significantly increased, and the proportion of G2 phase cells was significantly decreased after ADCY1 knockdown, which means the proliferation activity of the cells was enhanced by the down-regulation of ADCY1. In other words, the knockdown of ADCY1 can reverse the inhibition of cell proliferation caused by cisplatin.

Then, we up-regulated the expression of ADCY1 in A549/DDP cells; we added cisplatin and cultured the cells for 48 h. The cell cycle distribution was detected by flow cytometry. As shown in Figure 4C, after we transferred the ADCY1 plasmid, the proportion of cells in the G2 phase of A549/DDP cells significantly increased, and the proportion of cells in the G1 phase significantly decreased, which can result in G2 phase arrest and cell proliferation inhibition.

### 2.5. The Downstream Genes of ADCY1 Screened by RNA Sequencing

**Figure 5:**
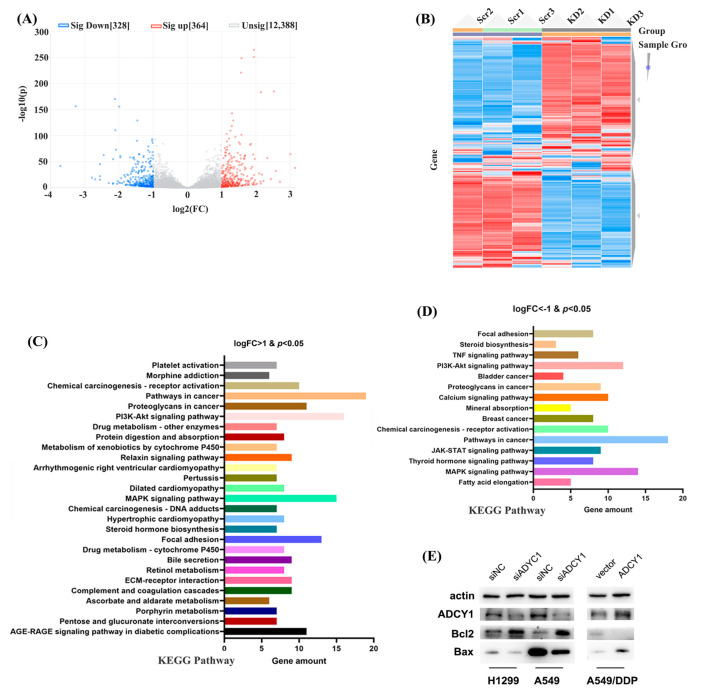
The downstream genes of ADCY1 were screened by RNA sequencing in A549 cells. (**A**) The volcano figure of downstream genes. (**B**) The heat map figure of downstream genes. (**C**) The KEGG pathway of logFC > 1 and *p* < 0.05 genes. (**D**) The KEGG pathway of logFC < −1 and *p* < 0.05 genes. (**E**) The regulation of Bcl2 and Bax by knockdown of the expression of ADCY1 in A549 and H1299 cells, and the up−regulation of the expression of ADCY1 in A549−DDP cells.

To further investigate the mechanism by which ADCY1 regulates platinum sensitivity, we down-regulated the expression of ADCY1 in A549 cells, repeated this three times, and then performed RNA sequencing. As shown in Figure 5A–D, triplicate knockdown of ADCY1 in A549 cells resulted in 328 co-down-regulated genes and 364 co-up-regulated genes. We also carried clustering analysis to obtain the heat map. Kyoto Encyclopedia of Genes and Genomes (KEGG) pathway analysis was also performed on genes regulated by ADCY1 to obtain the pathways related to ADCY1. It has been reported that B-cell lymphoma-2 (Bcl2) and Recombinant Bcl2-associated X Protein (Bax) are important markers of apoptosis. Bcl2 can control the endogenous apoptotic pathway, Bax is regulated by Bcl2, and Bax promotes apoptosis. As shown in Figure 5E, after the knockdown of ADCY1, compared with the control group, the expression of Bcl2 was increased and the expression of Bax was decreased in both A549 and H1299 cells, which was consistent with the decreased level of cell apoptosis. After the overexpression of ADCY1, the expression of Bcl2 was decreased and the expression of Bax was increased in A549/DDP cells compared with the control group, which was consistent with the increased level of cell apoptosis. We also performed gene ontology (GO) enrichment, including GO_BP, GO_CC, and GO_MF in the down-regulated genes and up-regulated genes, respectively (Figure S1, Supporting Information).

## 3. Discussion

In our previous study, we performed whole genome exon sequencing of blood samples in 17 NSCLC patients treated with platinum-based chemotherapy who were extremely sensitive and 17 patients who were extremely resistant to cisplatin. We found that ADCY1 RS2293106 (C > A) had a significant effect on the response of platinum-based chemotherapy in NSCLC patients, and patients carrying the A allele had a better response to platinum-based chemotherapy than those carrying the G allele. At the same time, it is also predicted that this site may affect the expression of ADCY1 through the binding of miRNA and mRNA [37]. In this study, we wanted to investigate the mechanism by which ADCY1 affects platinum-based chemotherapy response in NSCLC.

It has been reported that ADCY1 can induce β-cell dysfunction in pancreatic cancer; it is a significant diagnostic and prognostic biomarker for pancreatic adenocarcinoma [24,38]. So, we wanted to discuss the association between ADCY1 and lung cancer. First, we searched the cancer genome atlas (TCGA) database for the expression of ADCY1 mRNA in lung cancer tissues. It showed that the expression of ADCY1 was significantly higher in cancer tissues than adjacent cancer tissues in lung squamous carcinoma patients; however, there is no significant difference in the adenocarcinoma of the lung cancer patients. Interestingly, in the prognostic analysis, the low expression of ADCY1 is significantly beneficial to the prognosis of patients with lung adenocarcinoma; however, there is no significant difference in lung squamous carcinoma patients. This may be caused by the sample size not being large enough, indicating that ADCY1 still has potential research value and may be a prognostic biomarker for lung cancer patients treated with platinum-based chemotherapy.

As we all know, disease biomarkers always have a differential expression between cancer cells and normal cells [39]. The Survival assay is an intuitive detection method to identify cell sensitivity to cisplatin [40], which means that the higher the IC50 value, the more resistant it is to cisplatin [41]. We detected the mRNA level and protein expression of ADCY1 in MRC5 and lung cancer cells (A549, H1299, A549/DDP), which showed significant differences between the MRC5 cell and lung cancer cells. We knocked down the expression of ADCY1 in A549 and H1299 cells, overexpressed ADCY1 in A549/DDP cells, treated them with the cisplatin concentration gradient, and detected the IC50 value by MTT. The results showed that the low expression level of ADCY1 can lead to platinum resistance, and the IC50 value increased significantly. The high expression level of ADCY1 results in sensitivity to platinum and a significant decrease in the IC50 value. Combined with the fact that the expression of ADCY1 in cisplatin-resistant cells was lower than that in non-resistant cells, this means that the expression of ADCY1 may play an important role in cisplatin sensitivity.

It has been reported that cell proliferation and apoptosis are essential factors in the process of platinum resistance. The p53-related signaling pathway may participate in the process of apoptosis and reverse the process of cisplatin resistance through cell proliferation and apoptosis in non-small cell lung cancer cells [42]. It can promote cell proliferation, migration, and cell cycle activity, inhibit cancer cell apoptosis through the p53-mediated signaling pathway, and regulate platinum resistance in cervical cancer [43]. We investigated the effect of ADCY1 on lung cancer cell proliferation and apoptosis using CCK8 and Annexin V/propidium iodide (PI) assays, using the growth curves of cells, and using flow cytometry. The results showed that, over time, the growth of the ADCY1 knockdown group was faster than that of the control group, which demonstrated that the expression of ADCY1 was significantly associated with cell proliferation. Furthermore, cellular apoptosis was significantly reduced in ADCY1 knocked-down cells, and it was significantly increased after the overexpression of ADCY1. This means that ADCY1 plays an important role in cell apoptosis.

Although cisplatin is a non-specific drug for the cell cycle, it can also affect the progress of the cell cycle and lead to cell cycle arrest [44]. Reverse phase protein microarray identified up-regulated proteins in the resistant cell lines that are involved in apoptosis, cell proliferation, and cell cycle activity, which may provide a new therapeutic strategy for the treatment of SCLC [45]. We knocked down ADCY1 in A549 and H1299 cells, overexpressed it in A549/DDP cells, and detected cell cycle activity by flow cytometry. The results showed that cisplatin could induce G2 phase arrest and inhibit cell cycle progression. Compared with the control group, the proportion of S-phase cells was significantly increased and the proportion of G2 phase cells was significantly decreased in the knockdown group, which promoted the progress of the cell cycle. This was consistent with the result of promoting cell proliferation by knocking down the expression of ADCY1. Furthermore, after up-regulating the expression of ADCY1, the proportion of cells in the G2 phase was significantly increased, while the proportion of cells in the G1 phase was significantly decreased, and the cell cycle arrest in the G2 phase was not able to proceed, which was consistent with the result of inhibiting proliferation.

In order to find the downstream regulatory genes of ADCY1, we designed an RNAseq assay by knocking down the ADCY1 in A549 cells three times. We found 328 co-down-regulated genes and 364 co-up-regulated genes. We performed KEGG pathway and GO enrichment analyses in these differential genes. Among the differential genes, the RAC1 in the MAPK signaling pathway and ERBB3 in the MAPK signaling pathway caught our eyes. RAC1 and ERBB3 have been reported to be associated with drug resistance in lung cancer. Further research may be designed on the interaction between ADCY1 and RAC1/ERBB3. It has been reported that Bcl2 and Bax are the classical genes of cell apoptosis [46,47]. Bcl2 and Bax antiapoptotic proteins are often overexpressed in malignant cells [48]. We detected the expression of classical apoptotic pathway proteins Bcl2 and Bax. We found that after knockdown of ADCY1, the expression of Bcl2 was significantly increased and the expression of Bax was significantly decreased. While the overexpression of ADCYQ was contrary, the expression of Bcl2 was significantly decreased, and the expression of Bax was significantly increased. These results indicated that ADCY1 may regulate apoptosis by regulating Bcl2/Bax.

## 4. Materials and Methods

### 4.1. Materials

ADCY1 and β-actin antibodies were purchased from Sigma Aldrich (St. Louis, MO, USA). Cisplatin was obtained from Meilunbio (Dalian City, Liaoning Province, China). Cell culture media and reagents were from Corning (Corning, NY, USA) or Invitrogen (Carlsbad, CA, USA). Scramble siRNA and ADCY1 siRNA were purchased from RiboBio (Guangzhou City, Guangdong Province, China).

### 4.2. Cell Culture and siRNA Transfection

The A549, H1299, and SK-MES-1 cell lines were purchased from the Chinese Academy of Sciences (Shanghai, China) (https://www.cellbank.org.cn/, accessed on 16 May 2020), and the MRC5, H520, and A549/DDP cell lines were obtained from the cell biology research laboratory and Modern Analysis Testing Center of Central South University (Changsha, China). H1299, A549, A549/DDP, SK-MES-1, and HBE were cultured in RPMI-1640 medium supplemented with 10% FBS. MRC-5 was cultured in MEM medium supplemented with 10% FBS. All the cells were maintained at 37 °C in a humidified 5% CO_2_ atmosphere. Moreover, the A549/DDP cell line was cultured in medium with 2 mg/L cisplatin (Sigma, St. Louis, MO, USA, P4394) to maintain the drug-resistant phenotype before experimentation. For siRNA transfection, A549 and H1299 cells were seeded in 6-well plates and transfected with 80 nmol/L ADCY1 siRNA by using Lipofectamine RNA-iMAX and scramble siRNA was transfected as control. The cells were incubated for 48 h and we changed the medium to RPMI-1640 medium with 10% fetal bovine serum 6 h after transfection.

### 4.3. Plasmid Overexpression

A549/DDP cells were seeded in 6-well plates and transfected with a mixture of 1 μg plasmid ADCY1 and 2 μL P3000 reagent by using Lipofectamine 3000, and the vector was transfected as control. The cells were incubated for 6 h, then we changed the medium to α-MEM medium with 10% fetal bovine serum, and the cells were collected 48 h after transfection.

### 4.4. Real-Time Reverse Transcriptase PCR

The cells were extracted for total DNA with Trizol, Isopropanol, and chloroform with the following steps: First, 1 μg of total RNAs was reverse transcribed into gDNA by using a PrimeScript RT Reagent Kit with gDNA Eraser (Takara, Otsu City, Shiga Prefecture, Japan). Real-time quantitative PCR was analyzed by a LightCyder 480II system (Roche, Basel, Switzerland, Diagnostics) using the SYBR Premix Dimer Eraser Kit (Takara, Otsu City, Shiga Prefecture, Japan). The mRNA expressions of ADCY1, OGG1, APEX1, FANCI, APC11, CUL1, NFKB1, and PSME3 were analyzed for Ct value and β-actin as internal controls.

### 4.5. Western Blotting

For Western blotting, cells were lysed in RIPA buffer (Beyotime, China) containing PMSF (Beyotime, Haimen City, Jiangsu Province, China) and DTT (Beyotime, Haimen City, Jiangsu Province, China) at 4 °C for 30 min, then centrifuged at 12,000× *g* for 15 min at 4 °C. The total protein content was determined with a BCA Protein Concentration Assay Kit (Beyotime, China). Proteins transferred to a PVDF membrane (Millipore, St. Louis, MO, USA) were separated by SDS-PAGE. Then, the membrane was blocked using 5% nonfat dry milk for 1 h and incubated with primary antibodies at 4 °C overnight. Finally, the membrane was incubated by HRP-conjugated secondary antibodies for 1 h and the stripes were analyzed by ECL substrates and the ChemiDoc imaging system (Bio-Rad, Hercules, CA, USA).

### 4.6. Survival Assay

The influence of the expression of the ADCY1 gene on cisplatin drug resistance was analyzed by using the MTT assay. A549 and H1299 cells were transfected with ADCY1-siRNA/NC-siRNA in 6-well plates when cells had about 70% density and were planted into 96-well plates (4000 cells/well) for culturing for 24 h. Subsequently, the cells were treated with different concentrations of cisplatin by 48 h in a concentration gradient of 0.390625, 0.78125, 1.5625, 3.125, 6.25, 12.5, 25, 50, 100 μM. Finally, the medium was replaced with RPMI-1640 medium containing CCK-8 (CCK-8: RPMI-1640 = 1:9) to achieve a final concentration of 1 mg/mL. The plates were in a dark environment and incubated at 37 °C for 1–2 h; the absorbance was measured at 450 nm by using a microplate reader (Bio-Rad, CA, USA). GraphPad Software (version 9, San Diego, CA, USA) was used to plot the dose–response curve and calculate the half maximal inhibitory concentration (IC50) quantitative values.

### 4.7. Apoptosis Detection

According to the results of the MTT assay, H1299 cells, A549 cells, and A549 DDP cells were treated with si-ADCY1 or ADCY1 overexpression plasmid and then treated with cisplatin for 48 h. The apoptosis rate was evaluated using an Annexin V-FITC/PI Apoptosis Detection kit as the instruction. The cells were seeded into plates and resuspended in 500 μL binding buffer. Then, 5 μL Annexin V-FITC and 5 μL PI were added to the plates and incubated at 37 °C for 15 min in darkness. Annexin V-FITC is green fluorescence and propidium iodide (PI) is red fluorescence.

### 4.8. Cell Cycle Detection

Cells were seeded in a tissue culture plate and were collected into a 15 mL centrifuge tube. After the PBS washing treatment, 1 mL DNA Staining solution and 10 μL Permeabilization solution were added and incubated for 30 min at room temperature. The red fluorescence was detected at the excitation wavelength of 488 nm using a flow cytometer, and Cell Quest software (https://www.bdbiosciences.com/zh-cn, Becton Dickinson, Franklin Lakes, NJ, USA) was used for DNA content analysis and light scattering analysis. The percentage of cells in each phase was evaluated.

### 4.9. Statistical Analysis

All experimental data were analyzed by the computer software IBM SPSS Statistics 26.0 and graphed by GraphPad Prism 8. The experiment was repeated three times, and the data were expressed as mean ± standard error. Statistical differences in data were calculated using Student’s *t*-test or a paired *t*-test; *p* < 0.05 was considered to be statistically significant.

## 5. Conclusions

ADCY1 has a higher expression in lung cancer cells than human embryonic lung fibroblast cells (MRC5). Down-regulating the expression of ADCY1 can increase cisplatin resistance in lung cancer cells through the regulation of the promotion of cell proliferation and the inhibition of cell apoptosis; it can also affect cell cycle activity. ADCY1 may control cell apoptosis by regulating the classical apoptosis biomarkers Bax and Bcl2. It may also interact with RAC1 and ERBB3 in the MAPK signaling pathway, which needs further investigation.

## Data Availability

The supporting data related to our findings throughout our study, including the transcriptional data, will be available from the corresponding author [cj1028@csu.edu.cn] upon reasonable request.

## Supplementary Materials

The following supporting information can be downloaded at: https://www.mdpi.com/article/10.3390/ph17091118/s1, Figure S1: The downstream genes of ADCY1 screened by RNA sequencing in A549 cells by GO enrichment.
