# Supplementary material for: The Role of ADCY1 in Regulating the Sensitivity of Platinum-Based Chemotherapy in NSCLC
Source: Pharmaceuticals (Basel). 2024 Aug 24;17(9):1118. doi: 10.3390/ph17091118 (PMC11434658; doi:10.3390/ph17091118)
Supplement: Supplementary file 1 [file pharmaceuticals-17-01118-s001.zip › pharmaceuticals-3126817-supplementary.pdf]

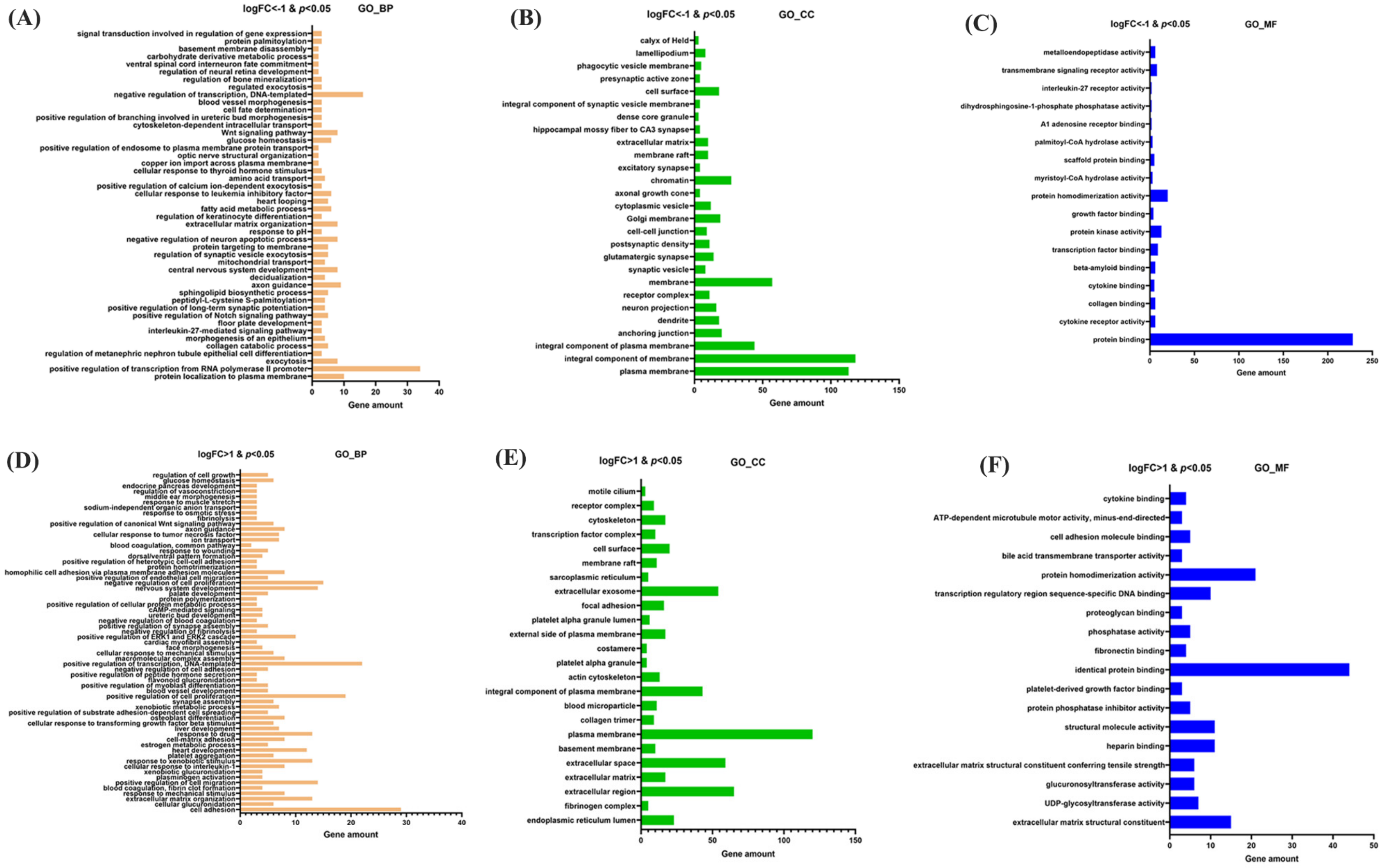
(A)
logFC<-1 & p<0.05
GO_BP
signal transduction involved in regulation of gene expression
protein palmitoylation
basement membrane disassembly
carbohydrate derivative metabolic process
ventral spinal cord interneuron fate commitment
regulation of neural retina development
regulation of bone mineralization
regulated exocytosis
negative regulation of transcription, DNA-templated
blood vessel morphogenesis
cell fate determination
positive regulation of branching involved in ureteric bud morphogenesis
cytoskeleton-dependent intracellular transport
Wnt signaling pathway
glucose homeostasis
positive regulation of endosome to plasma membrane protein transport
optic nerve structural organization
copper ion import across plasma membrane
cellular response to thyroid hormone stimulus
amino acid transport
positive regulation of calcium ion-dependent exocytosis
cellular response to leukemia inhibitory factor
heart looping
fatty acid metabolic process
regulation of keratinocyte differentiation
extracellular matrix organization
response to pH
negative regulation of neuron apoptotic process
protein targeting to membrane
regulation of synaptic vesicle exocytosis
mitochondrial transport
central nervous system development
decidualization
axon guidance
sphingolipid biosynthetic process
peptidyl-L-cysteine S-palmitoylation
positive regulation of long-term synaptic potentiation
positive regulation of Notch signaling pathway
floor plate development
interleukin-27-mediated signaling pathway
morphogenesis of an epithelium
collagen catabolic process
regulation of metanephric nephron tubule epithelial cell differentiation
exocytosis
positive regulation of transcription from RNA polymerase II promoter
protein localization to plasma membrane
0 10 20 30 40
Gene amount
(B)
logFC<-1 & p<0.05
GO_CC
calyx of Held
lamellipodium
phagocytic vesicle membrane
presynaptic active zone
cell surface
integral component of synaptic vesicle membrane
dense core granule
hippocampal mossy fiber to CA3 synapse
extracellular matrix
membrane raft
excitatory synapse
chromatin
axonal growth cone
cytoplasmic vesicle
Golgi membrane
cell-cell junction
postsynaptic density
glutamatergic synapse
synaptic vesicle
membrane
receptor complex
neuron projection
dendrite
anchoring junction
integral component of plasma membrane
integral component of membrane
plasma membrane
0 50 100 150
Gene amount
(C)
logFC<-1 & p<0.05
GO_MF
metalloendopeptidase activity
transmembrane signaling receptor activity
interleukin-27 receptor activity
dihydrosphingosine-1-phosphate phosphatase activity
A1 adenosine receptor binding
palmitoyl-CoA hydrolase activity
scaffold protein binding
myristoyl-CoA hydrolase activity
protein homodimerization activity
growth factor binding
protein kinase activity
transcription factor binding
beta-amyloid binding
cytokine binding
collagen binding
cytokine receptor activity
protein binding
0 50 100 150 200 250
Gene amount
(D)
logFC>1 & p<0.05
GO_BP
regulation of cell growth
glucose homeostasis
endocrine pancreas development
regulation of vasoconstriction
middle ear morphogenesis
response to muscle stretch
sodium-independent organic anion transport
response to osmotic stress
fibrinolysis
positive regulation of canonical Wnt signaling pathway
axon guidance
cellular response to tumor necrosis factor
ion transport
blood coagulation, common pathway
response to wounding
dorsal/ventral pattern formation
positive regulation of heterotypic cell-cell adhesion
protein homotrimerization
homophilic cell adhesion via plasma membrane adhesion molecules
positive regulation of endothelial cell migration
negative regulation of cell proliferation
nervous system development
palate development
protein polymerization
positive regulation of cellular protein metabolic process
cAMP-mediated signaling
ureteric bud development
negative regulation of blood coagulation
positive regulation of synapse assembly
negative regulation of fibrinolysis
positive regulation of ERK1 and ERK2 cascade
cardiac myofibril assembly
face morphogenesis
cellular response to mechanical stimulus
macromolecular complex assembly
positive regulation of transcription, DNA-templated
negative regulation of cell adhesion
positive regulation of peptide hormone secretion
flavonoid glucuronidation
positive regulation of myoblast differentiation
blood vessel development
positive regulation of cell proliferation
synapse assembly
xenobiotic metabolic process
positive regulation of substrate adhesion-dependent cell spreading
osteoblast differentiation
cellular response to transforming growth factor beta stimulus
liver development
response to drug
cell-matrix adhesion
estrogen metabolic process
heart development
platelet aggregation
response to xenobiotic stimulus
cellular response to interleukin-1
xenobiotic glucuronidation
plasminogen activation
positive regulation of cell migration
blood coagulation, fibrin clot formation
response to mechanical stimulus
extracellular matrix organization
cellular glucuronidation
cell adhesion
0 10 20 30 40
Gene amount
(E)
logFC>1 & p<0.05
GO_CC
motile cilium
receptor complex
cytoskeleton
transcription factor complex
cell surface
membrane raft
sarcoplasmic reticulum
extracellular exosome
focal adhesion
platelet alpha granule lumen
external side of plasma membrane
costamere
platelet alpha granule
actin cytoskeleton
integral component of plasma membrane
blood microparticle
collagen trimer
plasma membrane
basement membrane
extracellular space
extracellular matrix
extracellular region
fibrinogen complex
endoplasmic reticulum lumen
0 50 100 150
Gene amount
(F)
logFC>1 & p<0.05
GO_MF
cytokine binding
ATP-dependent microtubule motor activity, minus-end-directed
cell adhesion molecule binding
bile acid transmembrane transporter activity
protein homodimerization activity
transcription regulatory region sequence-specific DNA binding
proteoglycan binding
phosphatase activity
fibronectin binding
identical protein binding
platelet-derived growth factor binding
protein phosphatase inhibitor activity
structural molecule activity
heparin binding
extracellular matrix structural constituent conferring tensile strength
glucuronosyltransferase activity
UDP-glycosyltransferase activity
extracellular matrix structural constituent
0 10 20 30 40 50
Gene amount

Figure S1. The downstream genes of ADCY1 screened by RNA sequencing in A549 cells by GO enrichment. (A) The GO enrichment in GO_BP of logFC>1 & $p<0.05$ genes. (B) The GO enrichment in GO_CC of logFC>1 & $p<0.05$ genes. (C) The GO enrichment in GO_MF of logFC>1 & $p<0.05$ genes. (D) The GO enrichment in GO_BP of logFC<1 & $p<0.05$ genes. (E) The GO enrichment in GO_CC of logFC<1 & $p<0.05$ genes. (F) The GO enrichment in GO_MF of logFC<1 & $p<0.05$ genes.
